# Accuracy of immediate antepartum ultrasound estimated fetal weight and its impact on mode of delivery and outcome - a cohort analysis

**DOI:** 10.1186/s12884-018-1772-7

**Published:** 2018-05-02

**Authors:** Johannes Stubert, Adam Peschel, Michael Bolz, Änne Glass, Bernd Gerber

**Affiliations:** 1 Department of Obstetrics and Gynecology, University of Rostock, Suedring 81, 18059 Rostock, Germany; 2 Department of Radiology, Hospital Asklepios Klinik Barmbek, Hamburg, Germany; 3 Institute for Biostatistics and Informatics in Medicine, University of Rostock, Rostock, Germany

**Keywords:** Prenatal ultrasonography, Fetal biometry, Fetal weight estimation, Birth weight, Accuracy

## Abstract

**Background:**

The aim of the study was to investigate the accuracy of ultrasound-derived estimated fetal weight (EFW) and to determine its impact on management and outcome of delivery.

**Methods:**

In this single-center cohort analysis, women with a singleton term pregnancy in the beginning stages of labor were included. Women with immediately antepartum EFW (*N* = 492) were compared to women without ultrasound (*N* = 515).

**Results:**

EFW was correct (deviation from birth weight ≤ 10%) in 72.2% (355/492) of patients with fetal biometry; 19.7% (97/492) were underestimated, and 8.1% (40/492) were overestimated. Newborns with a lower birth weight were more frequently overestimated, and newborns with higher birth weight were more frequently underestimated. The mean difference between EFW and real birth weight was − 114.5 g (standard deviation ±313 g, 95% confidence interval 87.1–142.0). The rate of non-reassuring fetal heart tracing (9.8% vs. 1.9%, *P* < 0.001) and of caesarean delivery (9.1% vs. 5.0%, *P* = 0.013) was higher in women with EFW. Overestimation was associated with an increased risk for delivery by caesarean section (odds ratio 2.80; 95% confidence interval 1.2–6.5, *P* = 0.017). After adjustment, EFW remained associated with increased non-reassuring fetal heart tracing (odds ratio 4.73; 95% confidence interval 2.3–9.6) and caesarean delivery (odds ratio 1.86; 95% confidence interval 1.1–3.1). The incidence of perineal tears of grade 3/4, shoulder dystocia, postnatal depression and neonatal acidosis did not differ between groups.

**Conclusions:**

Antepartum ultrasound-derived EFW does not improve maternal and fetal outcome and is therefore not recommended.

## Background

Usually, first presentation to the delivery room of pregnant women at term occurs with onset of regular contractions. Although ultrasound examination of the fetus during admission is not routinely recommended, it is a common practice in German hospitals. There may be various reasons for this approach. On the one hand it gives certain information about fetal position and placental location that may be of relevance for management of the delivery. Otherwise, especially residents can gain experience in performance of ultrasound. From own experience it can be stated that ultrasound-derived estimation of fetal weight (EFW) is nearly always part of such an examination. In that way, fetuses with suspicion of macrosomia will also be identified. Fetal macrosomia is a known risk factor for adverse obstetric outcome parameters, such as shoulder dystocia, failure of progression and third and fourth-degree perineal tears [1–3]. The performance of ultrasound-derived EFW at the beginning of birth is not part of the recommendations of the international guidelines. However, if fetal macrosomia is assumed, particularly > 4500 g and in combination with diabetes, a delivery by caesarean section should be considered to reduce the potential morbidity [4, 5]. Although there is only weak evidence for this approach, the determination of EFW could be of forensic relevance in cases of birth-related damage; subsequently, claims are continuously increasing in Germany and other countries [6]. Consequently, knowledge of EFW could become legally relevant, and it could therefore influence the management of delivery and facilitate decisions in favor of a secondary caesarean section even if a vaginal delivery was initially intended. The aim of this study was to correlate the immediate antepartum ultrasound-derived EFW with the birth weight and to investigate if knowledge of EFW influences a) the management of delivery and b) maternal and fetal outcome parameters.

## Methods

### Trial design and participants

The study was performed at the Department of Obstetrics and Gynecology of the University of Rostock between May 2012 and February 2013. Written informed consent was obtained from all participants. A total of 1007 women with an uncomplicated singleton pregnancy and onset of regular contractions between 37 + 0 and 41 + 0 weeks of gestation were included. The exclusion criteria were premature onset of labor, multiple pregnancies, preterm membrane rupture, cervix dilatation > 5 cm, planned primary caesarean delivery, non-vertex presentation and suspected intrauterine growth restriction. The trial meets the criteria of a quasi-randomized design. The condition of the cervix in all the women was classified by a modified Bishop-Score [7]. Mothers with a mature cervix (score > 12) were directly prepared for delivery without ultrasound (*N* = 492); otherwise (score ≤ 12), ultrasound with EFW was additionally performed (*N* = 515). All women progressed spontaneously to the active phase of the first stage of labor without pharmacological or mechanical techniques of cervical ripening. In all cases, delivery was within seven days after EFW.

### Outcome measures

EFW was calculated using fetal abdomen circumference, the length of the femur and the biparietal diameter according to the formula of Hadlock II [8]. GE Logiq P6 (GE Medical Systems, Milwaukee, WI, USA) was used for sonographic examination. Residents had > 1 year of experience in ultrasound examination, and specialists had > 5 years of experience. Gestational age was calculated from the first day of the last menstrual period and was corrected by ultrasound if measurements of the crown-rump-length during the first trimester were different after more than 7 days. Intrapartum assessment was based on continuous fetal heart rate monitoring with a classification of heart rate patterns according to the FIGO-guidelines. The appraisal of cardiotocography and concomitant proceedings including fetal blood sampling, intrauterine resuscitation with β-mimetics or operative termination of pregnancy was decided by the specialist on duty. The following outcome parameters were registered: shoulder dystocia, third and fourth-degree perineal tears, neonatal depression (5’APGAR ≤7) and neonatal acidosis (umbilical arterial blood pH < 7.10 or base excess < − 10 mmol). Shoulder dystocia was assumed if a delayed development of the fetal shoulders required medical care by obstetric procedures.

### Statistical analysis

All data were stored and analyzed using the IBM SPSS statistical package 23.0 (SPSS Inc. Chicago, IL, USA) and Excel 2010 (Microsoft Corporation, Redmond, WA, USA). Descriptive statistics included mean and standard deviation (SD) for parametric as well as median and interquartile range (IQR) for non-parametric parameters. Frequency and relative percentage were used for categorical data. Testing for differences of continuous variables between groups was accomplished by the Student’s t-test or Mann-Whitney U-test as appropriate. Comparison of categorical variables between the groups was performed using the chi-square test or the Fisher’s exact test. *P* values resulted from two-sided statistical tests, and values < 0.05 were considered statistically significant. For the outcome parameters, caesarean section and non-reassuring fetal heart tracing odds ratios (ORs) were computed. Here, the logistic regression model was used to assess the independence of specific outcome parameters. In the multivariate model, the ORs were adjusted to maternal body mass index, nulliparity, gestational age at delivery, maternal weight gain during pregnancy, gestational diabetes, maternal age and birth weight. Correlations were computed using the Pearson’s correlation coefficient. The 95% confidence interval (CI) was reported to demonstrate the reliability of the estimated parameters. The percentage difference between EFW and real birth weight was calculated by the following formula: relative difference % = [(EFW - birth weight) / birth weight] × 100. A relative difference ± 10% was regarded as correct.

Receiver operating characteristic (ROC) curves were calculated for identification of hypertrophic and hypotrophic newborns by EFW and the areas under the curves (AUCs) were reported. The cut-off values were calculated for false positive rates of 5% and 10% and corresponding detection rate (DR) is given.

## Results

### Patients’ characteristics

The baseline characteristics of the included women were generally well balanced between both groups with only little differences (Table 1). The proportion of nulliparous women was slightly, but significant higher in the EFW group (58.5% vs. 52.2%; *P* = 0.049). Women who obtained EFW had also a higher mean weight gain during pregnancy (mean difference 0.79 kg, *P* = 0.049). Although mean gestational age at delivery was higher in women with EFW (*P* = 0.006), the mean difference was only two days. Inhomogeneity was also observed for the frequency of hypertensive disorders with more cases in women with EFW. Only one patient with hypertension got a caesarean section after EFW.

**Table 1:** Patient and fetal characteristics of study participants

Characteristic	Patients with EFW (*N* = 492)	Patients without EFW (*N* = 515)	p
Age, y	28.7 ± 5.2	28.7 ± 5.1	0.866^a^
Gravidity, n	2 (1–3)	2 (1–3)	0.478^b^
Parity, n	1 (1–2)	1 (1–2)	0.049^b^
Body mass index (pregravid), kg/m^2^	23.8 ± 4.8	23.6 ± 5.2	0.623^a^
Maternal weight gain in pregnancy, kg	15.92 ± 5.9	15.14 ± 6.1	0.049^a^
Gestational diabetes, n	32 (6.5%)	22 (4.3%)	0.116^c^
Hypertensive diseases, n	13 (2.6%)	3 (0.6%)	0.009^c^
Duration of the second stage, min	46.4 ± 50.0	46.0 ± 47.5	0.902^a^
Gestational age at delivery, d	280.2 ± 8.7	278.8 ± 7.8	0.006^a^
Birth weight, g	3528 ± 485	3540 ± 496	0.718^a^
Birth weight ≥ 4500 g, n	12 (2.4%)	16 (3.1%)	0.519^c^
Birth length, cm	50.4 ± 2.2	50.4 ± 2.1	0.795^a^
Head circumference, cm	35.1 ± 1.4	35.0 ± 1.4	0.386^a^
Head circumference ≥ 38 cm, n	20 (4.1%)	13 (2.5%)	0.160^c^
Umbilical arterial blood pH	7.27 ± 0.07	7.28 ± 0.07	0.378^a^
Base excess, mmol/L	− 4.64 ± 2.89	− 4.39 ± 2.84	0.167^a^
5´-APGAR-Score	10 (9–10)	10 (9–10)	0.695^b^

^a^ Student’s t-test for independent samples

^b^ Mann-Whitney U-test by ranks^c^ Pearson’s χ2-test for homogeneity

### Accuracy of fetal weight estimation

**Fig. 1:**
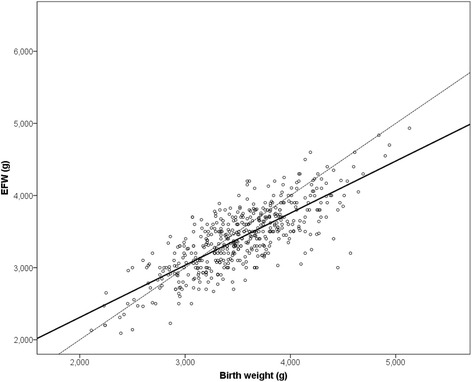
Scatter plot of neonatal birth weight and antepartum ultrasound-derived estimated fetal weight: The solid line represents the calculated linear regression with y = 0.722*x + 866 and a coefficient of determination R^2^ = 0.606. The dotted line represents the ideal regression with y = x. Newborns with lower birth weight were overestimated, and newborns with higher birth weight were underestimated by antepartum ultrasound

**Fig. 2:**
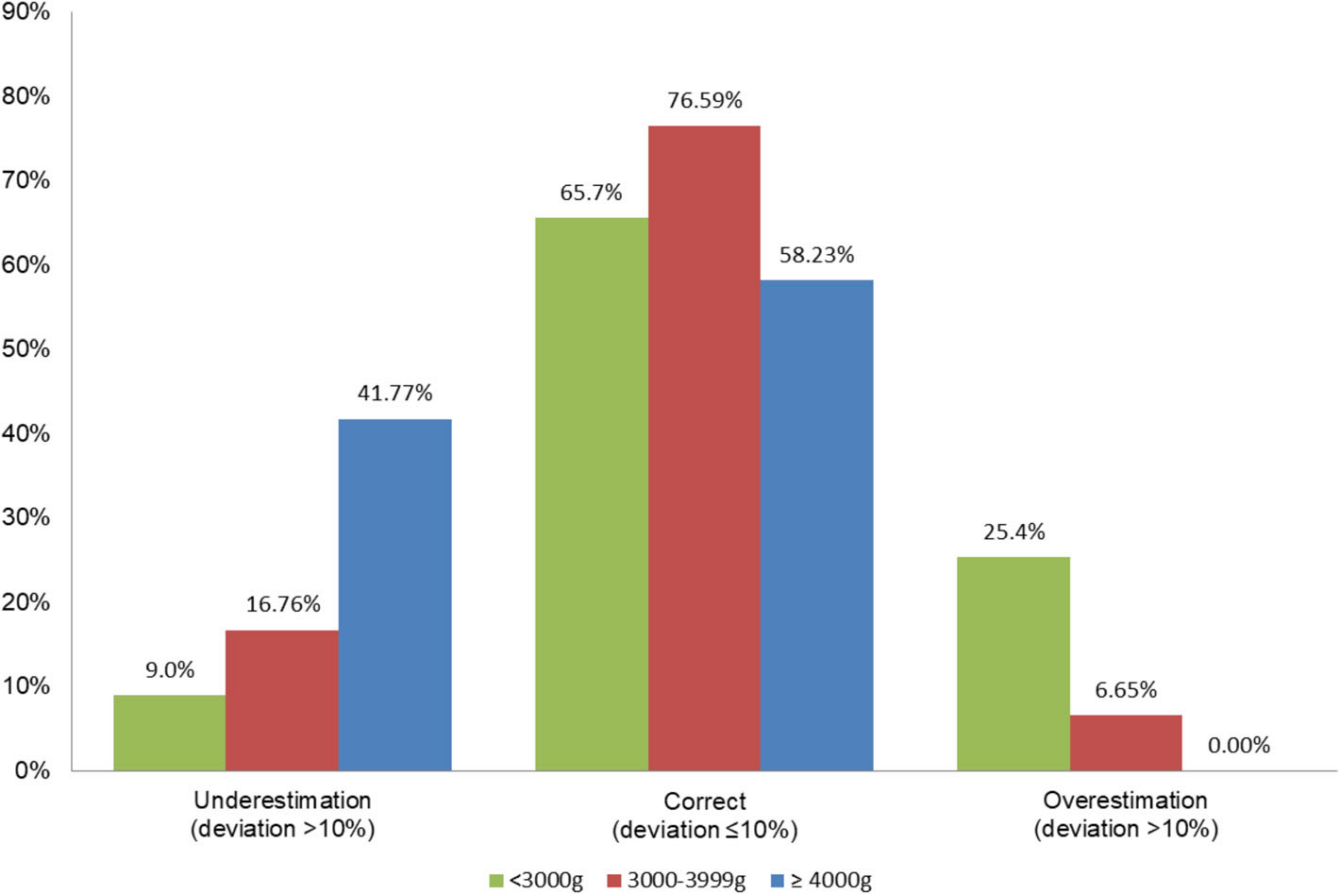
Accuracy of EFW in relation to the birth weight of the newborn. Accurate weight estimation was assumed when the deviation from birth weight was within ±10%. The best fit was achieved between 3000 and 3999 g. Hypertrophic newborns were more frequently underestimated, and hypotrophic newborns more frequently overestimated

Antepartum assumed EFW and real birth weight were well correlated with a Pearson’s correlation coefficient of *r* = 0.778 and a coefficient of determination of R^2^ = 0.606 (Fig. 1). In the entire group, mean birth weight was underestimated by − 113.6 g ± 313 g (95% CI -141.3 to − 85.9; *P* < 0.001), which correlates to a mean relative difference of − 2.75% ± 8.8% (95% CI -3.5 to − 2.0; *P* < 0.001). The absolute estimation error (sum of all difference values/n) was 261.5 g. Of all EFW, 72.2% were evaluated exactly with an underestimation of 19.7% and an overestimation of 8.1%. The accuracy depended on birth weight with an increase in overestimation at a birth weight < 3000 g and an increase in underestimation at a birth weight ≥ 4000 g (Fig. 2). Best performance was achieved in the subgroup of newborns with a birth weight between 3000 and 3900 g and ultrasound performed by specialists (*n* = 151): mean difference − 86.6 g ± 275 g (95% CI -130.8 to − 42.5, *P* < 0.001) and mean relative difference − 2.30% ± 7.9% (95% CI -3.6 to − 1.0, *P* < 0.001). The accuracy was 79.5% in this subgroup (74.0% for residents, 76.6% all investigators, *P* = 0.251). The accuracy between residents and specialists was also not different in the total study population (69.1% vs. 75.6%, *P* = 0.129). However, in the subgroup of newborns with a birth weight > 4000 g, the specialists conducted significantly more correct measurements compared to the residents (73.0% vs. 45.2%, *P* = 0.022). Relative differences between EFW and neonatal birth weight were neither correlated to pregravid maternal body mass index, maternal weight gain during pregnancy, parity nor to gestational age at delivery (all *P* > 0.05).

### Test characteristics for detection of hyper- and hypotrophic newborns

DRs for hypertrophia (birth weight ≥ 4500 g) were 83.3%, 58.3% and 41.7% at an EFW cut-off-level of ≥4000 g, 4300 g and 4500 g with the corresponding FPRs of 7.9%, 1.4% and 0.4%. ROC-analysis resulted in an AUC of 0.92 (95% CI 0.81 to 1.00, *P* < 0.001). Calculated DRs were 83% at a FPR of 10% (EFW cut-off 3900 g) and 67% at a FPR of 5% (EFW cut-off 4100 g).

DRs for hypotrophia (birth weight ≤ 2500 g) were 60.9% and 47.8% at an EFW cut-off-level of ≤2700 g and 2500 g with the corresponding FPRs of 2.8% and 0.9%. The AUC was 0.97 (95% CI 0.94–1.00, *P* < 0.001). Calculated DRs were both 83% at a FPR of 5% (EFW cut-off 2760 g) and at a FPR of 10% (EFW cut-off 2900 g).

### Mode of delivery and outcome parameters between groups

As shown in Table 2, women with antepartum EFW underwent caesarean section more frequently (9.1% vs. 5.0%, *P* = 0.013) with an adjusted OR of 1.86 (95% CI 1.1 to 3.1; Table 3). Among patients with caesarean delivery, we did not observe a difference in birth weight (3504 ± 667.9 g without and 3474 ± 671.9 g with EFW, *P* = 0.858). There were also no differences with respect to mean head circumference and maternal weight gain during pregnancy (data not shown). EFW did not differ between patients with and without caesarean delivery (*P* = 0.262).

**Table 2:** Mode of delivery, maternal and short-term fetal outcome parameters

Characteristic	Patients with EFW (*N* = 492)	Patients without EFW (*N* = 515)	P^*^
Caesarean delivery, n	45 (9.1%)	26 (5.0%)	0.013
Failure to progress, n	28 (5.7%)	17 (3.3%)	0.069
Nonreassuring fetal assessment	48 (9.8%)	10 (1.9%)	< 0.001
Shoulder dystocia	2 (0.4%)	2 (0.4%)	1.000
Perineal tear III°/IV°	1 (0.2%)	2 (0.4%)	1.000
5’APGAR ≤ 7	12 (2.4%)	13 (2.5%)	1.000
Umbilical arterial blood pH < 7.10	6 (1.2%)	3 (0.6%)	0.330
Base excess < −10 mmol/L	10 (2.0%)	7 (1.4%)	0.467

^*^ Fisher’s exact test

**Table 3:** Association between estimation of fetal weight and risk of caesarean delivery, nonreassuring fetal assessment and failure to progress. ORs were adjusted to maternal body mass index, nulliparity, gestational age at delivery, maternal weight gain during pregnancy, gestational diabetes, maternal age and birth weight

	Patients without EFW	Patients with EFW	P^*^	crude OR (95%CI)	adjusted OR (95%CI)
Caesarean delivery, n	26/515 (5.0%)	45/492 (9.1%)	0.013	2.80 (1.2–6.5)	1.86 (1.1–3.1)
Nulliparous women	18/269 (6.7%)	27/288 (9.4%)	0.278	1.44 (0.8–2.7)	1.41 (0.7–2.7)
Multiparous women	8/246 (3.3%)	18/204 (8.8%)	0.014	2.88 (1.2–6.8)	3.11 (1.3–7.8)
Nonreassuring fetal assessment, n	10/515 (1.9%)	48/492 (9.8%)	< 0.001	5.46 (2.7–10.9)	4.73 (2.3–9.6)
Nulliparous women	6/269 (2.2%)	36/288 (12.5%)	< 0.001	6.26 (2.6–15.1)	5.37 (2.2–13.2)
Multiparous women	4/246 (1.6%)	12/204 (5.9)	0.020	3.78 (1.2–11.9)	3.57 (1.1–11.6)
Failure to progress, n	17/515 (3.3%)	28/492 (5.7%)	0.069	1.77 (1.0–3.3)	1.59 (0.8–3.0)
Nulliparous women	12/269 (4.5%)	27/288 (9.4%)	0.030	2.21 (1.1–4.5)	2.00 (1.0–4.1)
Multiparous women	5/246 (2.0%)	1/204 (0.5%)	0.228	0.24 (0.0–2.5)	0.33 (0.0–3.2)

^*^ Fisher’s exact test

However, the accuracy of EFW was different between both groups. In patients who underwent a caesarean section, the EFW was significantly more frequently overestimated (17.8% vs. 7.2%, *P* = 0.013) with an OR of 2.80 (95% CI 1.2–6.5, *P* = 0.017). The rate of caesarean section did not differ significantly between classes of birth weight, but there was a trend in higher numbers of hypo- and hypertrophic newborns (16.4% < 3000 g, 7.5% 3000–3999 g and 10.1% ≥4000 g; *P* = 0.065).

Interestingly, non-reassuring fetal heart tracing was more common in women with EFW in the entire study group (9.8% vs. 1.9%, *P* < 0.001; adjusted OR = 4.73, 95% CI 2.3–9.6, *P* < 0.001) and also if only patients who underwent caesarean delivery were considered (31.1% vs. 7.7%, *P* = 0.037). Frequency of non-reassuring fetal heart tracing did not differ between patients with and without overestimation of EFW (8.3% vs. 8.1%, *P* = 0.957).

Patients with known EFW revealed a trend to a higher frequency of failure to progress (5.7% vs. 3.3%, *P* = 0.069). A similar trend was observed if only patients with overestimation of EFW were considered (17.9% vs. 7.5%, *P* = 0.067; OR = 2.67 (95% CI 0.9–7.4, *P* = 0.061), but differences were restricted to nulliparous women (Table 3).

Although knowledge of EFW increased the rate of caesarean section, the short term fetal and maternal morbidity was not improved in this group (Table 2). The results were also not significant even if we compared a composite morbidity endpoint including all single outcome parameters (13.3% vs. 30.8%, *P* = 0.075).

## Discussion

In accordance with numerous other studies, our results confirmed that the ultrasound-derived EFW during labor at term is an appropriate diagnostic tool, with an average accuracy of 70% within a relative difference of ±10% to the real birth weight [9–13]. We also observed a systematic underestimation of fetal weight in the total population. The frequency of underestimation was highest in newborns with a birth weight > 4000 g. In this subgroup, only 58% were correctly estimated, and none were overestimated. Therefore, EFW at term is of limited value for identification of fetal macrosomia. In our study, the accuracy did not depend on the pregravid maternal body mass index. However, there were only five women with a body mass index ≥40 in our study cohort. Other studies demonstrated a decrease of accuracy when the body mass index increased [14–17]. Although accuracy was nearly the same between residents and specialists in the total study population, specialists had more correct results when only newborns with a birth weight > 4000 g were considered.

In a postpartum study Kehl et al. directly measured the two-dimensional biometric parameters head circumference, abdominal circumference and thigh length (instead of the ultrasound parameter femur length) on 419 term newborns and computed the best-fitting formula for calculation of birth weight by a forward regression analysis [18]. Results were validated on validation group of further 209 newborns. With their new formulae a further increase of accuracy with avoidance of a systematic error was possible. They concluded that a good sonographic weight formula should have an accuracy of 80% within a discrepancy level of 10% with a SD of about 7% and without a systematic error. However the new formulae also revealed the problem of a general overestimation of birth weight in the lower weight range and an underestimation at the upper end of the range. Furthermore, as the authors circumvented the performance of ultrasound they did not consider the influence of measurement errors resulting from oligohydramnios, thick abdominal wall, deep pelvic head position and inaccuracy in measurement of the abdominal circumference. In a recent study Eggebø et al. demonstrated that it is possible to achieve the quality of fetal weight estimation postulated by Kehl and colleagues [19]. In this study the ultrasound examination was performed on 419 women on day 290 of pregnancy. With an algorithm including gestational age the authors reported of an accuracy of 83% within 10% discrepancy, a SD of 7.6% and without a systematic error (mean difference between birth weight and FWF was -6 g). Even if these results were impressive, the detection rates for macrosomia and small for gestational age fetuses were only 54% and 49% on false positive rate of 5%. In comparison, sensitivity was not superior to our results (using the less accurate formula of Hadlock II) for detection of the cases of most clinical importance.

Consequently, EFW at term is not reliable for prediction of macrosomia and is therefore not recommended by several guidelines. Taking the international guidelines into account, the rate of caesarean delivery should not be higher if fetal weight was estimated immediately before delivery. However, in the present study, it was nearly doubled. The increase was independent of fetal weight, and it was not restricted to macrosomic fetuses. However, the overestimation of fetal weight was associated with an increased risk for caesarean delivery. In a retrospective cohort analysis, EFW was also associated with an increased risk for caesarean delivery (OR 1.44, 95% CI 1.1–1.9) [20]. Similar results were found in a recently published big cohort study of 64,030 women at term who attempted vaginal delivery [21]. In this study the knowledge of EFW was significantly associated with an increased risk of caesarean delivery (adjusted OR 1.44 (95% CI 1.31–1.58, *P* < 0.001). In contrast to our results, several studies correlated the risk of caesarean delivery with an increase of the EFW > 3500 g [20–22]. Our data supported the study results of Blackwell et al., which showed that overestimation of fetal weight (in contrast to absolute weight estimation) was an independent risk factor for caesarean delivery (OR 4.8, 95% CI 1.5–15.2) [23]. In a further retrospective cohort analysis, the overestimation of large gestational age fetuses was identified as a risk factor for caesarean delivery in newborns with birth weight between 2500 and 3499 g (OR 2.82, 95% CI 1.62–4.84, *P* < 0.01) as well as 3500–4500 g (OR 3.47, 95% CI 2.06–5.88, *P* < 0.01) [24]. So there is rising evidence, that knowledge of EFW by itself is a risk factor for decision to a caesarean delivery.

In our study, the increase of the caesarean delivery rate was neither accompanied by a decrease of fetal nor maternal morbidity. In particular, no differences in shoulder dystocia and third and fourth degree perineal tears were observed. In a case-control study of 1938 women with antenatal EFW eight cases of shoulder dystocia were observed [22]. In four cases dystocia occurred in women with EFW < 4000 g. Although fetal macrosomia is a known risk factor for the development of shoulder dystocia, 20–65% of all cases of shoulder dystocia occur in children with a birth weight below 4000 g [25]. In a recent study by Peleg et al. on newborns with birth weight > 4000 g (238 non-diabetic low risk women with EFW ≥4000 g and 205 women with EFW < 4000 g), the risk of caesarean delivery was 9.0-times higher when macrosomia was correctly assumed, but there was no difference in the rate of shoulder dystocia [26]. Overall, there is strong evidence that EFW increases the rate of caesarean delivery with no impact on the rate of shoulder dystocia [27–32].

In the EFW group, a non-reassuring fetal heart tracing was diagnosed more often, although we found no differences in short-term fetal outcome. This is a new observation that is difficult to explain. We hypothesize that the obstetricians were hypercritical when interpreting the fetal heart rate patterns, and they were seeking an indication to perform caesarean delivery following the overestimation of fetal weight.

The strengths of this study are its prospective, quasi-randomized design, which maintained mostly well balanced patient characteristics between both groups. The availability of outcome parameters allowed statements regarding not only the accuracy of EFW and its influence on the mode of delivery but also regarding the fetal and maternal morbidity.

A potential bias in our results may be founded by the different maturity of the cervix at inclusion. Several studies observed a correlation between cervical dilatation at admission and the risk of a caesarean section [33–37]. These studies compared patients with cervical dilatation of 0–3 cm to patients with dilatation of 4 to 10 cm. Earlier admission to delivery room was associated with an increased risk of a caesarean delivery. However, essential differences to our study are obvious. First, we included only patients during the latent phase of labor with cervical dilatation < 5 cm [38]. Second, as even postulated in some of these studies, the observed increase of caesarean delivery was probably caused by physician intervention, e.g. augmentation of labor with oxytocin [34, 35, 37]. In contrast, in our study intervention for cervical ripening or labor augmentation was avoided during the latent phase of labor. At least, our observed differences between patients with and without EFW were confirmed by the increased risks for caesarean section in patients with overestimation of EFW. In this inner group comparison a bias related to criterions of inclusion can be clearly excluded.

However, it cannot be ruled out, that the differences of cervical maturity between our groups may have an influence on the frequency of caesarean section. Further limitations of our study are the limited patient numbers and the generally low number of outcome events. Hence, we presented a composite outcome. There were some additional imbalances between the groups in terms of parity, maternal weight gain during pregnancy, gestational age at delivery and the frequency of hypertensive disorders (which were generally low); therefore, we computed a logistic regression analysis by adjusting for these possible confounding variables.

## Conclusion

Antepartum ultrasound derived EFW is, although widely used, of limited clinical benefit. Its accuracy substantially decreases in the detection of hypo- and hypertrophic fetuses. The overestimation of fetal weight correlates with an increased risk of caesarean delivery. Nevertheless, antepartum ultrasound-derived EFW does not improve maternal and fetal outcome and is therefore not recommended.

## Abbreviations

AUC: area under the curve
CI: confidence interval
DR: detection rate
EFW: estimated fetal weight
FPR: false positive rate
IQR: interquartile range
OR: odds ratio
ROC: receiver operating characteristic
SD: standard deviation
